# Effectiveness of Physical Therapy Interventions for Temporomandibular Disorders Associated with Tinnitus: A Systematic Review

**DOI:** 10.3390/jcm12134329

**Published:** 2023-06-28

**Authors:** Marianne Trajano da Silva, Carlos Silva, Jade Silva, Mateus Costa, Inae Gadotti, Karyna Ribeiro

**Affiliations:** 1 Post-Graduation Program of Physical Therapy, Federal University of Rio Grande do Norte, Natal 59078-970, RN, Brazil; maritrj23@gmail.com; 2 Department of Physical Therapy, Federal University of Rio Grande do Norte, Natal 59078-970, RN, Brazil; carloshpestana96@gmail.com (C.S.); jadepadilha@gmail.com (J.S.); mateusantonio09@gmail.com (M.C.); 3 Department of Physical Therapy, Florida International University, Miami, FL 33199, USA; igadotti@fiu.edu

**Keywords:** temporomandibular joint, physical therapy modalities, tinnitus, pain

## Abstract

Temporomandibular disorders (TMDs) refers to different clinical conditions affecting the temporomandibular joints, masticatory muscles, and adjacent structures. Although TMDs signs and symptoms (e.g., pain and limited mouth opening) are common, otological symptoms, such as tinnitus, might also be present. This study aims to summarize the evidence of the effectiveness of physical therapy interventions in individuals with TMDs associated with tinnitus. Randomized controlled trials investigating the effectiveness of physical therapy in individuals of both genders aged 18 or older with TMDs associated with tinnitus were included. The electronic search was performed in the following databases: MEDLINE, EMBASE, CINAHL, PEDro and CENTRAL. A total of four studies were included. All studies showed that physical therapy reduced the intensity of tinnitus, and two trials showed a decrease in the pain intensity caused by TMDs, an increase in the pressure pain thresholds in the masticatory muscles and an improvement of mandibular function. Two studies presented a low risk of bias. Despite a low certainty of the evidence, this review showed that physical therapy reduces the intensity or severity of tinnitus associated with TMDs. Results may support future research on the topic and evidence-based practice by recommending the best physical therapy approach for patients, clinicians, researchers, and health system managers.

## 1. Introduction

Temporomandibular disorders (TMDs) refer to different clinical conditions affecting the temporomandibular joints (TMJ), the masticatory muscles, and adjacent structures [1]. The prevalence of TMDs is estimated to be 31% in adults/elderly and 11% in children/adolescents [2], and it affects more women than men [3]. The main symptom of TMDs is local or diffused orofacial pain. However, joint noises, limited mouth opening, neck pain, headache, and otologic symptoms (i.e., tinnitus, hyperacusis, vertigo, and dizziness) might also be present [4,5,6,7].

Tinnitus is defined as an ear or head perceived sound in the absence of external stimuli [8], and its prevalence ranges from 5% to 42% of the global population [9]. Tinnitus is commonly related to hearing loss, cochlear lesions, changes in the auditory nervous system, and somatosensory system including cervical spine disorders and TMJ [8,10]. The prevalence of tinnitus associated with TMDs ranges from 30.4% to 36.6% [11,12]. Patients with TMDs present an eight times greater risk of developing tinnitus when compared with a control group [11].

According to a systematic review and meta-analysis, patients with TMDs experience tinnitus more often than patients without TMDs [13]. In addition, an increase of TMJ related complaints from 19% to 36% was found in a group of subjects with severe tinnitus when compared to all levels of tinnitus [14], which suggest that TMJ pain, myalgia, presence of bruxism, popping, and mandibular dysfunction such as difficulty chewing, may contribute to tinnitus severity [14,15]. Therefore, an intervention targeting these signs and symptoms may benefit patients with tinnitus and TMJ complaints as they may be considered a separate subtype of tinnitus [15].

Although more studies are needed to better understand TMDs and somatosensory tinnitus and the pathophysiology behind it, the relationship between the cervical region and TMJ is well established. Asymmetry within the masticatory muscle activity may be related to the presence of active myofascial trigger points within the trapezius muscle, which suggest that the presence of myofascial pain in the cervical muscles may play a role in the disturbances of the stomatognathic system [16]. In addition, studies have demonstrated neuroanatomical connections among the cervical region, temporomandibular region, and cochlear nucleus. Somatosensory afferents from the upper cervical spine and TMJ merge into the medullary somatosensory nucleus, which projects nerve fibers into the dorsal cochlear nucleus. Disinhibition of the dorsal cochlear nucleus through the somatosensory pathway produces an excitatory neuronal activity in the auditory pathway that may be responsible for somatosensory tinnitus [10,16,17]. This hypothesis is supported by the increased activity of muscles innervated by the trigeminal nerve and the presence of trigger points in masticatory, cervical, and shoulder girdle muscles that may provoke or modulate tinnitus intensity [18,19]. Although not usually associated with masticatory muscles, tensor tympani and tensor veli palatini muscles may develop a reflex contraction and cause tinnitus. This may occur due to the tonic tensor tympani syndrome that results in hyperacusis symptoms, tinnitus, a sensation of ear fullness, dizziness, and TMJ pain [20]. A phylogenetic relationship between the anatomy of the auditory tube and TMJ is also present since they come from the same embryonic cartilage [21].

A multidisciplinary approach is important in the management of patients with TMDs associated with tinnitus since it has also an impact on quality of life and leads to sleep disturbances, anxiety, stress, and mood swings [14,22,23,24,25]. Evidence suggests that conservative treatment based on physical therapy improves symptoms and mandibular function in individuals with TMDs [25,26].

Two systematic reviews [27,28] investigated the effectiveness of conservative treatment (e.g., counseling, exercise, manual therapy, occlusal splint) exclusively in TMDs associated with tinnitus. However, these studies did not summarize the effectiveness of physical therapy, either isolated or associated with other therapies and included clinical trials and other types of study designs. Another recent review [29] evaluated the effect of manual therapy on pain pressure threshold and quality of life in patients with somatic tinnitus associated with the cervicogenic and temporomandibular dysfunction domain. These reviews did not provide information regarding specific criteria for the diagnosis of TMDs and the outcomes related to TMDs including mandibular dysfunction, TMJ pain, etc. Therefore, this systematic review aims to summarize the evidence of the effectiveness of different physical therapy interventions for individuals with TMDs associated with tinnitus regarding the intensity of pain, the pressure pain threshold of the masticatory and cervical muscles, and tinnitus intensity.

## 2. Materials and Methods

### 2.1. Protocol and Registration

This systematic review was registered in the International Prospective Register of Systematic Reviews (PROSPERO number: CRD42021228484). The selected methods followed the recommendations of Preferred Reporting Items for Systematic Reviews and Meta-Analyses (PRISMA) [30]. The research question for this systematic review followed the PICO strategy (Population, Intervention, Comparison, Outcomes): Population consisted of patients older than 18 years with TMDs and tinnitus; Intervention encompassed isolated physiotherapy or physiotherapy associated with other therapies; Comparison included placebo or no treatment; and outcomes consisted of pain intensity, pressure pain threshold, and tinnitus intensity and severity as primary outcomes, and mandibular incapacity, the influence of tinnitus, quality of life and adverse effects as secondary outcomes.

In this review, the following research question was answered: What is the effectiveness of isolated physical therapy interventions or combined with other therapies in treating patients with TMDs and associated tinnitus?

### 2.2. Eligibility Criteria

This study included randomized controlled trials, with participants aged 18 or older, from both genders, and with TMDs and tinnitus diagnoses. TMDs diagnosis had to be determined by the Research Diagnostic Criteria for Temporomandibular Disorders (RDC/TMD) [31] or Diagnostic Criteria for Temporomandibular Disorders (DC/TMD) [32]. Tinnitus diagnosis had to be determined by self-reporting data or complementary audiology exams (such as audiometry or acuphenometry) or both. Studies involving participants with a history of acute facial and cervical trauma, and neurologic diseases were excluded.

As for the interventions, primary studies that investigated relevant physical therapy interventions of any duration, frequency, or intensity to treat patients with TMDs associated with tinnitus, such as manual therapy (soft tissue mobilization, joint mobilization, manipulation, or muscle energy techniques), therapeutic exercise (strengthening exercises, active range of motion exercises, stretching, and motor control), photobiomodulation (laser therapy), electrotherapy, dry needling, counseling, or patient education were included. Physical therapy interventions comparing themselves, placebo, no intervention, or physical therapy associated with other common treatments (e.g., occlusal splint, botulinum toxin, cognitive behavioral therapy, and acupuncture) were included.

The primary outcome was the intensity of TMDs pain measured by validated self-report scales, pressure pain threshold in the TMJ area, masticatory and cervical musculature measured by a pressure algometer, tinnitus intensity and severity measured by validated self-report scales. Secondary outcomes were mandibular incapacity assessed by specific and validated questionnaires, the influence of tinnitus on daily life assessed by validated and specific questionnaire to tinnitus, quality of life evaluated by validated self-report instruments and adverse effects.

There were no language or date restrictions. The search strategy used for each database is included in Supplementary Material 1.

### 2.3. Data Management and Selection of Studies

All search results were exported and uploaded to the software Mendeley Desktop (version 1.19.8) to remove duplicates. In addition, a selection of studies was performed using the Rayyan online literature management software (https://rayyan.qcri.org, accessed on 1 November 2022). Three reviewers (CS, MS, and MC) independently conducted a review of the title and abstract of the selected articles, and the full text to identify the eligibility of the studies. The disagreements regarding the process selection of studies were solved by discussion and consulting a fourth author (KR).

### 2.4. Data Extraction

Before data extraction, a pilot test was performed to increase reliability between reviewers (MC, MS and JS). An extraction data form adapted from Cochrane Handbook [33] was used and three reviewers independently extracted the data and fulfilled the questionnaire for each study. In case of disagreement, a fourth author (KR) was consulted. Additional information or missing data was requested from the authors of included studies by email.

### 2.5. Assessment of the Risk of Bias in Included Studies

Three reviewers (MC, MS and JS) independently assessed the risk of bias in each included study using the PEDro scale. In case of disagreement, a fourth author (KR) was consulted. The PEDro scale is composed of 11 items with “yes” and “no” answer options. Item one refers to external validity, items two to nine refer to internal validity, and items 10 to 11 provide sufficient statistical information to interpret the results. Each “yes” answer consists of one point. The score is only assigned when the criteria are explicitly clear in the article (https://pedro.org.au, accessed on 1 December 2022). Since item one was not considered for the analysis, the maximal total score was 10 points.

### 2.6. Data Analysis

Due to the high heterogeneity among the interventions of the studies, it was not possible to perform a meta-analysis. However, the data were summarized using a narrative synthesis according to the interventions and outcomes. A forest plot was used to show the results of the studies and the direction of treatment effects visually. The Revman 5.4 software was used to build the forest plot for all the comparisons. The standardized mean difference (SMD) was used for analyzing continuous results.

The assessment of the certainty of the evidence was determined by Grading of Recommendations, Assessment, Development, and Evaluation (GRADE), which analyzes the following domains: study design, risk of bias, inconsistency, indirect evidence, imprecision, and other considerations (publication bias, large effect, confounding factors) [33]. The studies were classified as one of the four levels of certainty of evidence (high, moderate, low, or very low).

### 2.7. Patients and Public Involvement

A patient diagnosed with TMDs and tinnitus was selected and participated in the systematic review protocol to guide the development of the research question and the important outcomes based on the patient’s perspective. This procedure is based on the Guidance for Reporting Involvement of Patients and the Public (GRIPP) [34]. This step was conducted before the search strategy, in which a patient was recruited from a waiting list for physical therapy services in Rio Grande do Norte in Brazil.

The interview with the patient was conducted by video call. During the interview, the patient reported discomforts, such as pain in the masticatory and neck muscles, tinnitus, and “click sounds” close to the TMJ which is commonly observed in the population of interest for this study. According to the patient, the outcomes of interest that should be studied were the reduction of muscle pain, and the intensity and frequency of the tinnitus since it impairs daily functions and concentration/attention.

## 3. Results

### 3.1. Study Selection

**Figure 1:**
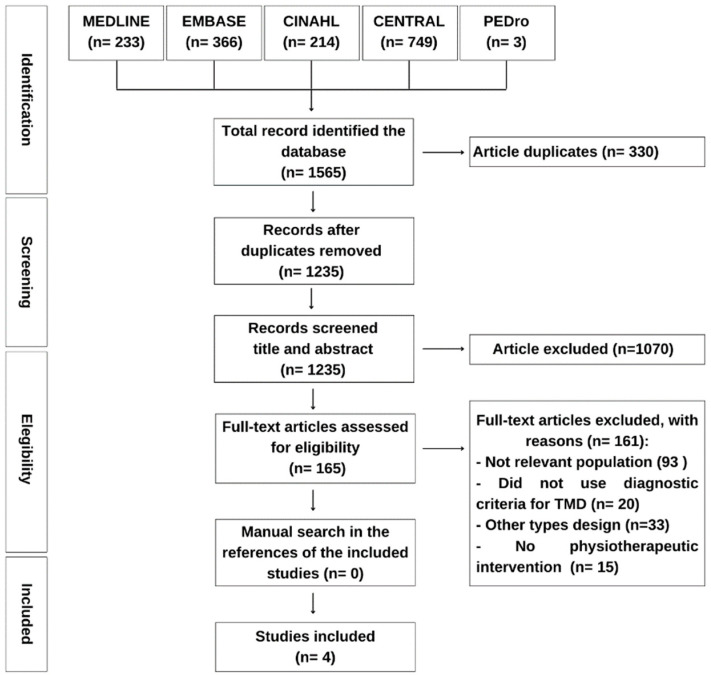
Flowchart of the study selection process.

The electronic search generated a total of 1565 articles. From those articles, only four were included [35,36,37,38] for summarizing the evidence (Figure 1). However, two articles are derived from the same study [35,37]. One of them [35] was a secondary analysis from the clinical trial of the La Serna et al. study [37].

The descriptive characteristics of the selected articles are presented in Table 1.

The number of participants per study ranged from 46 to 80 subjects, with a total of 187 individuals, of which 51.9% were women and 48.1% were men. The average age of the subjects in the studies was 42 years, ranging from 38 to 45 years. Three studies used the RDC/TMD for the diagnosis of TMDs, except one that adopted the DC/TMD [38]. As for the diagnosis of tinnitus symptoms, all of them applied self-reported tools.

Tinnitus severity, reported by mean annoyance and volume, was assessed by Visual Analog Scale (VAS) in two studies [35,37]. One of the studies [36] measured the tinnitus intensity using the VAS. In another study [38], tinnitus annoyance was evaluated using the Tinnitus Questionnaire (TQ), and the tinnitus severity was evaluated using the Tinnitus Functional Index (TFI). Both instruments applied are related to the tinnitus symptomatology but according to the authors, these instruments have different constructs and are more sensitive to differentiate tinnitus annoyance and intensity. The impact of tinnitus on daily life was assessed using the Tinnitus Handicap Inventory (THI) in two articles [35,37].

IG: Intervention Group. CG: Control Group. M: Men. W: Women. TMJ: Temporomandibular joint. SCOM: Sternocleidomastoid. NRPS: Numerical Pain Rating Scale. PPT: Pressure Pain Threshold. VAS: Visual Analog Scale. TQ: Tinnitus Questionnaire. TFI: Tinnitus Functional Index. THI: Tinnitus Handicap Inventory. CF-PDI: Craniofacial Pain and Disability Inventory. SF-12: Short Form Health Survey 12. Nd: YAG: neodymium-doped yttrium aluminum garnet. The intensity of TMDs pain was assessed using the Numerical Pain Rating Scale (NPRS) only in two articles [35,37]. These same studies also evaluated the Pressure Pain Threshold (PPT) over the lateral aspect of TMJ, and over the masseter and temporalis muscles using a digital algometer. The Craniofacial Pain and Disability Inventory (CF-PDI) was also used to measure incapacity, and the Short Form Healthy Survey 12 (SF-12) to measure the quality of life [35,37].

The studies clearly described their exclusion criteria. The most frequently applied criteria were individuals with neurological disorders, middle ear disease, Ménière’s disease, history of trauma or fractures in the head or neck region, severe depression or anxiety disorders, pregnancy, and previous treatment for orofacial pain in the last 3 to 12 months.

### 3.2. Interventions

The studies by La Serna et al. and Plaza-Manzano et al. [35,37] proposed education associated with mobility, posture, neck motor control, and manual therapy exercises (mandibular mobilization and soft tissue techniques for masticatory and cervical muscles). In the study by Van der Wal et al. [38], participants underwent an orofacial treatment consisting of counseling and other physical therapy modalities such as stretching, masticatory muscle massage, and relaxing therapy. In addition, for patients with cervical dysfunctions, exercises and mobilizations were added, and in cases of individuals with habits of teeth grinding, the use of an occlusal splint was recommended. Although most studies have proposed active therapies, only one study [36] used low-intensity laser in the auditory acoustic meatus when compared to the daily application of the Nd:YAG laser with the Diode, for a period of two weeks. The interventions are described in Table 1.

The duration of the treatment programs ranged from 2 to 9 weeks, with 6 to 18 sessions in total, and with an average of 11 sessions. Only two studies provided information regarding the duration of the session, which lasted about 30 min [35,37].

**Figure 2:**
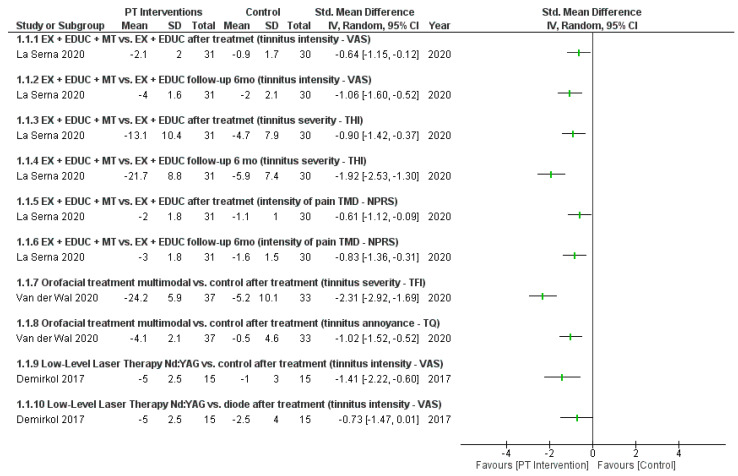
Forest plot for qualitative comparison of physical therapy interventions vs. other therapy or controls used in the analyzed studies. PT: Physical Therapy Interventions, VAS: Visual Analog Scale, THI: Tinnitus Handicap Inventory, NPRS: Numerical Pain Rating Scale, TFI: Tinnitus Functional Index, TQ: Tinnitus Questionnaire [35,36,38].

The description of the effects of the physical therapy interventions from all studies are shown in Figure 2. The study by La Serna et al. [35] showed a reduction of 21.7 points in the THI after intervention, while the study by Van der Wal et al. [38] showed a reduction of 24.2 points in the TFI. Furthermore, an improvement in the treatment effect on tinnitus intensity was observed in the study by La Serna et al. [35] after a six month follow-up.

### 3.3. Methodological Quality

**Figure 3:**
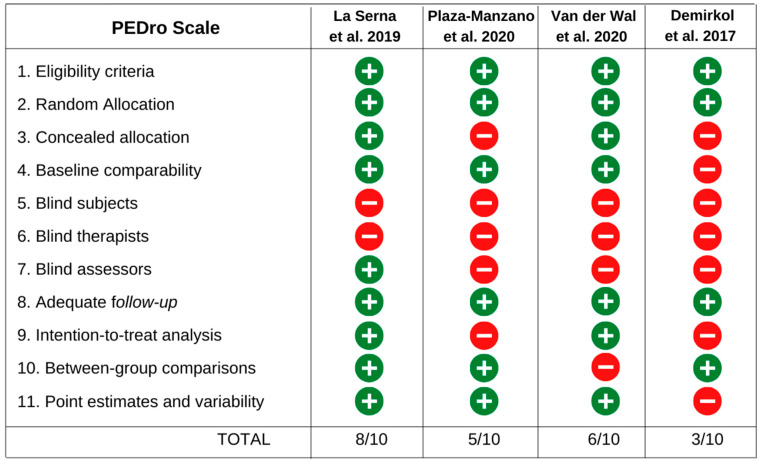
Evaluation of risk of bias for the studies included [35,36,37,38].

The PEDro scale was used to assess the methodological quality of the studies. The total score ranged from 3 to 8 points, with an average of 5.5 points. Two studies [35,38] showed high methodological quality. One study was rated as low methodological quality [36]. The scores of all studies are described in Figure 3. Of the four studies included, only two performed an intention-to-treat analysis for at least one of the outcomes [35,38].

The certainty of the evidence was assessed using the GRADE. One study was classified as having a moderate level of evidence [35]. The other two studies [36,38] remained in the low level evidence category. The downgraded was done for two reasons: the high risk of bias and the problem of imprecision. The certainty of the evidence of the studies is specified in Table 2, Table 3 and Table 4.

## 4. Discussion

This systematic review aimed to summarize the evidence of the effectiveness of physical therapy interventions for individuals with TMDs associated with tinnitus. Physical therapy can provide great benefits for patients with TMDs associated with tinnitus by treating the symptoms and improving quality of life [25]. The studies showed proportional amounts regarding the sex distribution of the subjects included, although TMDs are more prevalent in women [3]. The mean age was 42 years old, slightly higher than the age range most often affected by this condition [39].

All studies included the assessment of tinnitus symptoms to measure the effects of proposed physical therapy interventions. The VAS was the most used tool to assess tinnitus, which corroborates with the current literature [40,41]. This tool is shown to have good validity and reliability for the evaluation of tinnitus and is considered easy to use [42]. Other tools such as TQ, TFI [38] and THI [35,37] were also used to measure symptoms of tinnitus. In addition to evaluating the intensity of tinnitus, two studies [35,37] evaluated the TMDs pain intensity as a primary outcome. These trials also included other outcome measurements such as PPT of the masticatory muscles and TMJ, mandibular incapacity and quality of life [35,37].

Demirkol et al. [36] applied two modalities of low-level laser therapy, Nd:YAG 1064 nm and diode 810 nm in individuals with chronic subjective tinnitus associated with TMDs. In this study, the tinnitus reduction was found only at the four-week follow-up for both laser therapy groups. The mechanisms of laser action in tinnitus are still discussed; however, the most accepted theory is that irradiation promotes an increase in blood flow in the inner ear and induces hair cell repair through mitochondrial activation [43]. Despite the positive result in this trial and the possible mechanism of action, a recent meta-analysis showed no significant improvement in tinnitus with laser therapy intervention [44].

Van der Wal et al. [38] proposed a conservative orofacial treatment for individuals with somatosensory tinnitus attributed to TMDs. This multimodal intervention included the physical therapy modalities of counseling, stretching, massage of the masticatory muscles, and relaxation therapy, and, in some cases, the occlusal splint was indicated. The patients who also suffered from cervical problems, received cervical mobilizations and exercises. They included a group receiving an early-start treatment and a group delayed-start. The early-start group presented a significant difference in the TFI score immediately after the treatment when compared to the delayed-start, which supports the recommendation to treat tinnitus patients early because they have a favorable prognosis [45]. The TFI is a questionnaire mostly used to measure the severity of tinnitus and it has been shown to be more sensitive than TQ to detect smaller differences in measurements [46]. This study conducted a delayed treatment design instead of using a control group that received no treatment. Both received the same multimodal therapy but at different time points (nine weeks). Additionally, more than half of the patients included in this study (52%) were allocated evenly in the groups by cluster randomization with the use of an occlusal splint due to the presence of teeth grinding [38]. Considering that this oral behavior is often related to the aggravation of TMDs and tinnitus [36], some studies include occlusal splint treatment for the management of TMDs, bruxism or teeth clenching. However, the literature is controversial and with low methodological quality, indicating that the isolated use of the occlusal splint is not recommended for TMDs patients [24,47].

La Serna et al. [35] applied manual therapy associated multimodal physical therapy treatment in patients with tinnitus attributed to TMDs. This intervention demonstrated positive effects by decreasing the severity of the tinnitus and the intensity of TMDs pain, by increasing PPT of the temporalis and masseter muscles, and by improving mandibular function, immediately after the intervention and after follow-up evaluations (three and six months) [35]. It is important to emphasize that this study was classified with the lowest risk of bias among all studies (Figure 3).

Plaza-Manzano et al. [37] performed a secondary analysis of predictive variables in the treatment of these individuals according to the therapy administered in the abovementioned study [35]. This study showed that higher scores of tinnitus severity at baseline predicted better outcomes at three and six months post-intervention in both groups. For the patients who underwent exercise/education plus manual therapy, those with higher scores for tinnitus-related handicap at baseline predicted better clinical outcomes of tinnitus related handicap. It seems easier to elicit greater changes in an outcome with higher baseline scores because subjects with less pain and disability had less room to exhibit improvements [37]. Low PPT in the temporalis muscles in both groups of intervention predicted poorer clinical outcomes in tinnitus severity and THI at three and six months. This would be a relevant finding from a clinical viewpoint, since early identification of peripheral sensitization (decreased PPTs) over the trigeminal area could lead to better outcomes by implementing early management in individuals with TMDs-related tinnitus. Moreover, men were shown to have worse Tinnitus Related-Handicap (THI) responses after three months of treatment compared to women [37]. These findings are contrary to the current literature on pain perception between genders, which shows that there are no differences between genders in most pain modalities [48].

The four studies included in this review investigated different physical therapy treatment modalities at different time points. Therefore, a forest plot for different subgroups was performed based on interventions and outcomes (Figure 2). Considering the studies that used VAS as a tool to measure tinnitus intensity [35,36,37], the greatest improvement observed was after the intervention using the Nd:YAG laser [36] with a 5-point improvement followed by exercise/education plus manual therapy intervention [35] with a 4-point improvement after six months follow-up. The interventions using exercise/education alone and the diode laser improved tinnitus intensity by only 2-points [35,36]. Demirkol et al. study [36] was classified as having a very low level of certainty of the evidence, while the study by La Serna et al. [35] was classified as having a moderate level of certainty. In Van der Wal et al. study [38], significant differences in tinnitus severity was reported between the early start and delayed start groups, but this was not observed for tinnitus annoyance. In addition, for the entire group, more clinically pronounced improvement in tinnitus severity (TFI) was reported in 41% of patients immediately after treatment, and in 61% after follow-up.

Furthermore, except for the Demirkol et al. study, the studies also presented follow-up data in addition to data immediately after the intervention [35,37,38]. Better improvement was at follow-ups. This was explained by the fact that patients were able to change their habits, continue the exercises, and were less focused on the tinnitus complaints after the study [38].

Manual therapy techniques are widely used and recommended within multimodal therapy for the management of craniocervical dysfunctions [49,50,51]. The mechanical stimulation of this intervention triggers a neurophysiological cascade in the central and peripheral nervous system, responsible for the reduction of muscle activity and pain inhibition [52]. The manual therapy techniques cause reactive hyperaemia or a spinal reflex mechanism for muscle spasm relief by equalizing the length of the sarcomeres in the involved muscle [53]. Both mechanisms may explain the pain relief caused by the use of manual therapy techniques [54]. The study by Van der Wal et al. [38] applied massage techniques on the masticatory muscles and also on the cervical region when subjects presented associated cervical dysfunction, while the study by La Serna et al. [35] used techniques of jaw joint and soft tissue mobilizations for the orofacial and cervical region. In addition, other authors [25] recommend approaches to the cervical region such as active or passive mobilization and motor control exercises for the improvement of TMDs patients. Corroborating the findings of these studies, a clinical trial with 33 patients performed trigger point inactivation and an improvement in tinnitus severity measured by general characteristics (duration, location, type, perception, intensity) and THI was observed [18]. The use of manual therapy was also shown to be effective in improving quality of life as well as PPT in patients with both cervicogenic somatic tinnitus and TMDs associated with somatic tinnitus [29].

Most studies used exercises as part of the intervention [35,37,38]. The literature suggests that exercise therapy leads to improved flexibility and tissue extensibility, as well as increased muscle activity and endurance, and pain relief [25,55]. However, the trials are heterogeneous regarding the modalities of the exercises used, and they do not provide clarity as to their prescription (frequency, intensity, and duration). Future studies should report more detailed information about the exercise implemented. This is also critical for the reproducibility of the studies.

The implementation of education or counseling techniques in multimodal treatment for the management of patients with TMDs and tinnitus is worth emphasizing, especially because high rates of chronicity were shown in this population [22,56]. La Serna et al. study [35] approached not only the classical concepts, but also current concepts based on the neuroscience of pain, with active strategies for the management of painful conditions, strategies to change habits and distraction techniques. These concepts have been highlighted by research in the field of pain as a change of paradigm from purely disease-centered education [57]. Regarding tinnitus, counseling is an essential therapeutic modality for the management of the symptoms because its action contributes to the habituation of the phantom noise [58]. Furthermore, this therapeutic modality favors the autonomy/self-efficacy of patients in managing and coping with symptoms.

The multimodal treatment for TMDs associated with tinnitus comprises an intervention branch, given the multifactorial causes of tinnitus and TMDs. The biopsychosocial conservative treatment, with a biobehavioral component, currently gains space when compared to the biomedical and invasive model, as scientific evidence also indicates the relationship between psychosocial factors and the perception of symptoms by the patient [25,57].

None of the studies included in this review provided information regarding the relationship between different TMDs subgroups and the occurrence of tinnitus, and which physical therapy intervention is most effective for each TMDs subgroup. This should be addressed in future studies as tinnitus signs and symptoms may differ according to different TMDs classifications.

Based on the four studies included in this review [35,36,37,38], there is a low level of evidence regarding the effectiveness of physical therapy for individuals with TMDs associated with tinnitus, which was attributed to the limited studies available and their limited methodological quality (risk of bias) (Figure 3). However, based on the results of the forest plot (Figure 2) and the certainty of the evidence, it is suggested that clinicians use additional manual therapy (soft tissue release of the masticatory and cervical muscles, and TMJ mobilization), counseling, exercises for temporomandibular and cervical regions, and cervical mobilizations (in cases of cervical dysfunction) in individuals with TMDs associated with tinnitus. The effectiveness of physical therapy modalities applied to these patients should be further investigated with higher methodological quality clinical trials with possible implementation of protocols with placebo interventions for better observation of the natural course of the condition.

The present review followed a previous elaborated and registered protocol, including only randomized controlled clinical trials and TMDs diagnosed only by the validated and reliable tools DC/TMD and RDC/TMD. In addition, the clinical perspective was considered, making the research question relevant to patients and clinicians. However, the review was limited by the small number and low methodological quality of the included studies, and further meta-analysis of the results was not possible to perform to compare the effect size and mean differences of each physical therapy modality due to the heterogeneity of the studies’s interventions.

## 5. Conclusions

Physical therapy interventions based on manual therapy associated exercises and counseling, treatment orofacial conservative and low- level laser therapy were effective to reduce the intensity or severity of tinnitus in patients with TMDs associated with tinnitus. However, the results are based on limited evidence. Further investigation of other impaired clinical parameters in these patients including orofacial and neck pain, incapacity, and quality of life is needed. Results will support future research on the topic and evidence-based practice by recommending the best physical therapy approach for patients, clinicians, researchers, and health system managers.

## Figures and Tables

**Table 1:** Descriptive characteristics of selected studies.

Study and Country	Sample	Average Age (years)	Intervention Group	Comparison Group	Frequency and Duration of Intervention	Outcomes and Evaluation Measures	Follow-Up	Results
La Serna et al. (2020) [35] Spain Plaza-Manzano et al. (2020) [37] Spain	GI: 31 (12 M e 19 W) GC: 30 (13 M e 17 W)	GI: 42.5 (±12.0) GC: 44 (±10.5)	Education based on pain mechanisms, coping strategies and changing habits. Mobility, posture, TMJ motor control, tongue and neck exercises. Mandibular mobilization and soft tissue techniques in the massater, temporalis, SCOM and upper trapezius.	Education based on pain mechanisms, coping strategies and changing habits. Mobility, posture, TMJ motor control, tongue and neck exercises.	6 sessions of 30 min in 4 weeks.	Intensity of TMDs pain (NPRS), PPT (algometry), tinnitus severity (VAS), tinnitus report (THI), incapacity (CF-PDI) and quality of life (SF-12).	3 months 6 months	The exercise/education plus manual therapy group showed better outcomes in intensity of TMDs pain, tinnitus severity, PPT and incapacity. The follow-up three and six months theses outcomes proved significantly better. However, similar changes for the quality of life as the exercise/education alone group. In Both groups of treatment higher scores of tinnitus severity predicted better outcomes 3 and 6 months pos-intervention (*p* = 0.001). In addition, lower PPTs in the temporalis muscle predicted poorer clinical outcomes (*p* < 0.005). In group with manual therapy higher scores THI predicted better outcomes post-intervention (*p* < 0.005).
Van der Wal et al. (2020) [38] Belgium	GI: 40 (18 M e 22 W) GC: 40 (24 M e 16 W)	GI: 46 (±13) GC: 45 (±15)	Counselling, stretching, masticatory muscle massage and relaxation therapy. For patients with cervical dysfunctions, mobilizations and exercises for the region were added (early-started group).	Participants remained on the waiting list for 9 weeks, after which they also underwent the same intervention (delayed-started group).	18 sessions in 9 weeks.	Tinnitus annoyance (TQ) and tinnitus severity (TFI).	9 weeks 18 weeks	There was a clinically important and statistically significant (*p* < 0.005) reduction in the TQ and TFI score immediately after the intervention and at follow-up.
Demirkol et al. (2017) [36] Turkey	GI: 15 (7 M e 8 W) GC: 16 (10 M e 6 W) GP: 15 (6 M e 9 W)	GI: 36.6 (±14. 7) GC: 40.1 (±14.6) GP: 37.7 (±13.8)	Application of the Nd:YAG laser, 20 s continuously in the external acoustic meatus, with output power of 0.25 W, pulse duration 1000 ls (VLP mode), frequency of 10 Hz, pulse energy of 25 mJ, 25 W of power of peak and energy density of 8 J/cm^2^.	Application of 810 mn diode laser, 9 s continuously in the external acoustic meatus, with energy density of 8 J/cm^2^ and output power of 0.25 W. Placebo laser was applied anteromedially to the external acoustic meatus, without irradiation.	10 sessions in 2 weeks.	Tinnitus intensity (VAS)	1 month	In the two groups that received the laser, there was a reduction in tinnitus intensity after one month of treatment (*p* < 0.001). The group receiving Nd:YAG showed greater percentage improvement in tinnitus severity (*p* < 0.001).

**Table 2:** Manual therapy, exercise and education compared to exercise and education in patients with TMDs associated with tinnitus.

Certainty Assessment							No. of Patients		Effect		Certainty
No. of Studies	Study Design	Risk of Bias	Inconsistency	Indirectness	Imprecision	Other Considerations	Manual Therapy, Exercise and Education	Exercise and Education	Relative (95% CI)	Absolute (95% CI)	
**Intensity of pain after treatment (follow-up: mean 6 months; assessed with NPRS: scale from 0 to 10)**											
1	Randomized trials	Not serious	Not serious	Not serious	Serious ^a^	None	30	31	-	SMD 0.61 SD lower (1.12 lower to 0.09 lower)	⊕⊕⊕◯ Moderate
**Tinnitus intensity after treatment (follow-up: mean 6 months; assessed with VAS: scale from 0 to 10)**											
1	Randomized trials	Not serious	Not serious	Not serious	Serious ^a^	None	30	31	-	SMD 0.64 SD lower (1.15 lower to 0.12 lower)	⊕⊕⊕◯ Moderate
**Tinnitus severity after treatment (assessed with THI: scale from 0 to 100)**											
1	Randomized trials	Not serious	Not serious	Not serious	Serious ^a^	None	31	30	-	SMD 0.9 SD lower (1.42 lower to 0.37 lower)	⊕⊕⊕◯ Moderate

CI: confidence interval; SMD: standardised mean difference; a. The total of participants in comparison this lower than the optimal information size; ⊕⊕⊕◯: moderate level of certainty of evidence.

**Table 3:** Should Orofacial treatment multimodal compared to control in patients with TMDs associated with tinnitus.

Certainty Assessment							No. of Patients		Effect		Certainty
No. of Studies	Study Design	Risk of Bias	Inconsistency	Indirectness	Imprecision	Other Considerations	Orofacial Treatment Multimodal	Control	Relative (95% CI)	Absolute (95% CI)	
**Tinnitus annoyance after treatment (assessed with TQI: scale from 0 to 84)**											
1	Randomized trials	Serious	Not serious	Not serious	Serious ^a^	None	33	33	-	SMD 1.02 SD lower (1.52 lower to 0.52 lower)	⊕⊕◯◯ Low
**Tinnitus severity after treatment (assessed with TFI: scale from 0 to 100)**											
1	Randomized trials	Serious	Not serious	Not serious	Serious ^a^	None	33	33	-	SMD 2.31 SD lower (2.92 lower to 1.69 lower)	⊕⊕◯◯ Low

CI: confidence interval; SMD: standardised mean difference; a. The total of participants in comparison this lower than the optimal information size; ⊕⊕◯◯: low level of certainty of evidence.

**Table 4:** Low-Level Laser Therapy Nd:Yag compared to Control in patients with TMDs associated with tinnitus.

Certainty Assessment							No. of Patients		Effect		Certainty
No. of Studies	Study Design	Risk of Bias	Inconsistency	Indirectness	Imprecision	Other Considerations	Low-Level Laser Therapy Nd:Yag	Control	Relative (95% CI)	Absolute (95% CI)	
**Tinnitus intensity (follow-up: median 1 months; assessed with VAS: scale from 0 to 10)**											
1	Randomized trials	Serious	Not serious	Not serious	Serious ^a^	None	15	15	-	SMD 1.41 SD lower (2.22 lower to 0.6 lower)	⊕⊕◯◯ Low

CI: confidence interval; SMD: standardised mean difference; a. The total of participants in comparison this lower than the optimal information size; ⊕⊕◯◯: low level of certainty of evidence.

## Data Availability

The data presented in this study are available on request from the corresponding author. The data are not publicly available due to data protection issues.

## Supplementary Materials

The following supporting information can be downloaded at: https://www.mdpi.com/article/10.3390/jcm12134329/s1, Supplementary Material 1: the search strategy used for each database.

## References

[1] Ohrbach R., Dworkin S.F. (2019). AAPT Diagnostic Criteria for Chronic Painful Temporomandibular Disorders. J. Pain.

[2] Valesan L.F., Da-Cas C.D., Réus J.C., Denardin A.C.S., Garanhani R.R., Bonotto D., Januzzi E., de Souza B.D.M. (2021). Prevalence of temporomandibular joint disorders: A systematic review and meta-analysis. Clin. Oral Investig..

[3] Bagis B., Ayaz E.A., Turgut S., Durkan R., Özcan M. (2012). Gender Difference in Prevalence of Signs and Symptoms of Temporomandibular Joint Disorders: A Retro-spective Study on 243 Consecutive Patients. Int. J. Med. Sci..

[4] Conti P.C.R., Pinto-Fiamengui L.M.S., Cunha C.O., Conti A.C.d.C.F. (2012). Orofacial Pain and Temporomandibular Disorders: The Impact on Oral Health and Quality of Life. Braz. Oral Res..

[5] Costa Y.M., Conti P.C.R., de Faria F.A.C., Bonjardim L.R. (2017). Temporomandibular Disorders and Painful Comorbidities: Clinical Association and Underlying Mechanisms. Oral Surg. Oral Med. Oral Pathol. Oral Radiol..

[6] Silveira A., Gadotti I.C., Armijo-Olivo S., Biasotto-Gonzalez D.A., Magee D. (2015). Jaw Dysfunction Is Associated with Neck Disability and Muscle Tenderness in Subjects with and without Chronic Temporomandibular Disorders. BioMed Res. Int..

[7] Porto De Toledo I., Stefani F.M., Porporatti A.L., Mezzomo L.A., Peres M.A., Flores-Mir C., De Luca Canto G. (2017). Prevalence of Otologic Signs and Symptoms in Adult Patients with Temporomandibular Disorders: A Systematic Review and Me-ta-Analysis. Clin. Oral Investig..

[8] Baguley D., McFerran D., Hall D. (2013). Tinnitus. Lancet.

[9] McCormack A., Edmondson-Jones M., Somerset S., Hall D. (2016). A Systematic Review of the Reporting of Tinnitus Prevalence and Severity. Hear. Res..

[10] Levine R.A., Nam E.C., Oron Y., Melcher J.R. (2007). Evidence for a Tinnitus Subgroup Responsive to Somatosensory Based Treatment Modalities. Prog. Brain Res..

[11] Buergers R., Kleinjung T., Behr M., Vielsmeier V. (2014). Is There a Link between Tinnitus and Temporomandibular Disorders?. J. Prosthet. Dent..

[12] Manfredini D., Olivo M., Martini A. (2015). Prevalence of Tinnitus in Patients with Different Temporomandibular Disorders Symptoms. Int. Tinnitus J..

[13] Bousema E.J., Koops E.A., van Dijk P., Dijkstra P.U. (2018). Association Between Subjective Tinnitus and Cervical Spine or Temporomandibular Disorders: A Systematic Review. Trends Hear..

[14] Vielsmeier V., Strutz J., Kleinjung T., Schecklmann M., Kreuzer P.M., Land-grebe M., Langguth B. (2012). Temporomandibular Joint Disorder Complaints in Tinnitus: Further Hints for a Putative Tinnitus Subtype. PLoS ONE.

[15] Edvall N.K., Gunan E., Genitsaridi E., Lazar A., Mehraei G., Billing M., Tull-berg M., Bulla J., Whitton J., Canlon B. (2019). Impact of Temporomandibular Joint Complaints on Tinnitus-Related Distress. Front. Neurosci..

[16] Ginszt M., Szkutnik J., Zieliński G., Bakalczuk M., Stodółkiewicz M., Litko-Rola M., Ginszt A., Rahnama M., Majcher P. (2022). Cervical Myofascial Pain Is Associated with an Imbalance of Masticatory Muscle Activity. Int. J. Environ. Res. Public Health.

[17] Shore S., Zhou J., Koehler S. (2007). Neural Mechanisms Underlying Somatic Tinnitus. Prog. Brain Res..

[18] Rocha C.B., Sanchez T.G. (2012). Efficacy of Myofascial Trigger Point Deactivation for Tinnitus Control. Braz. J. Otorhinolaryngol..

[19] Rocha C.A.C., Sanchez T.G., Tesseroli De Siqueira J.T. (2008). Myofascial Trigger Point: A Possible Way of Modulating Tinnitus. Audiol. Neurotol..

[20] Westcott M., Sanchez T., Diges I., Saba C., Dineen R., McNeill C., Chiam A., O’Keefe M., Sharples T. (2013). Tonic Tensor Tympani Syndrome in Tinnitus and Hyperacusis Patients: A Multi-Clinic Prevalence Study. Noise Health.

[21] Parker W.S., Chole R.A. (1995). Tinnitus, Vertigo, and Temporomandibular Disorders. Am. J. Orthod. Dentofac. Orthop..

[22] Mottaghi A., Menéndez-Díaz I., Cobo J.L., González-Serrano J., Cobo T. (2019). Is There a Higher Prevalence of Tinnitus in Patients with Temporomandibular Disorders? A Systematic Review and Meta-Analysis. J. Oral Rehabil..

[23] Vielsmeier V., Kleinjung T., Strutz J., Bürgers R., Kreuzer P.M., Langguth B. (2011). Tinnitus with Temporomandibular Joint Disorders: A Specific Entity of Tinnitus Patients?. Otolaryngol.-Head Neck Surg..

[24] Gauer R.L., Semidey M.J. (2015). Diagnosis and Treatment of Temporomandibular Disorders. Am. Fam. Physician.

[25] Gil-Martinez A., Paris-Alemany A., López-de-Uralde-Villanueva I., La Touche R. (2018). Management of Pain in Patients with Temporomandibular Disorder (TMD): Challenges and Solutions. J. Pain Res..

[26] McNeely M.L., Armijo Olivo S., Magee D.J. (2006). A Systematic Review of the Effectiveness of Physical Therapy Interventions for Temporomandibular Disorders. Phys. Ther..

[27] Feitoza C.C., de Lemos Menezes P. (2019). Prognosis of Conservative Treatment in Individuals with Temporomandibular Disorders and Tinnitus: A Systematic Review. Muscle Ligaments Tendons J..

[28] Michiels S., Nieste E., Van de Heyning P., Braem M., Visscher C., Topsakal V., Gilles A., Jacquemin L., De Hertogh W. (2019). Does Conservative Temporomandibular Therapy Affect Tinnitus Complaints? A Systematic Review. J. Oral Facial Pain Headache.

[29] Sharma P., Singh G., Kothiyal S., Goyal M. (2022). Effects of Manual Therapy in Somatic Tinnitus Patients Associated with Cervicogenic and Temporomandibular Dysfunction Domain: Systematic Review and Meta Analysis of Randomized Controlled Trials. Indian J. Otolaryngol. Head Neck Surg..

[30] Moher D., Liberati A., Tetzlaff J., Altman D.G., Altman D., Antes G., Atkins D., Barbour V., Barrowman N., Berlin J.A. (2009). Preferred Reporting Items for Systematic Reviews and Meta-Analyses: The PRISMA Statement. PLoS Med..

[31] Dworkin S.F., LeResche L. (1992). Research Diagnostic Criteria for Temporomandibular Disorders: Review, Criteria, Examinations and Specifications, Critique. Craniomandib. Disord..

[32] Schiffman E., Ohrbach R., Truelove E., Look J., Anderson G., Goulet J.-P., List T., Svensson P., Gonzalez Y., Lobbezoo F. (2014). Diagnostic Criteria for Temporomandibular Disorders (DC/TMD) for Clinical and Research Applications: Recommendations of the International RDC/TMD Consortium Network* and Orofacial Pain Special Interest Group†. J. Oral Facial Pain Headache.

[33] Higgins J.P.T., Green S. (2011). Cochrane Handbook for Systematic Reviews of Innervations. https://training.cochrane.org/handbook.

[34] Staniszewska S., Brett J., Simera I., Seers K., Mockford C., Goodlad S., Altman D.G., Moher D., Barber R., Denegri S. (2017). GRIPP2 Reporting Checklists: Tools to Improve Reporting of Patient and Public Involvement in Research. BMJ.

[35] Delgado de la Serna P., Plaza-Manzano G., Cleland J., Fernández-de-las-Peñas C., Martín-Casas P., Díaz-Arribas M.J. (2020). Effects of Cervico-Mandibular Manual Therapy in Patients with Temporomandibular Pain Disorders and Associated Somatic Tinnitus: A Randomized Clinical Trial. Pain Med..

[36] Demirkol N., Usumez A., Demirkol M., Sari F., Akcaboy C. (2017). Efficacy of Low-Level Laser Therapy in Subjective Tinnitus Patients with Temporomandibular Disorders. Photomed. Laser Surg..

[37] Plaza-Manzano G., Delgado-de-la-Serna P., Diaz-Arribas M.J., Rodrigues-de-Souza D.P., Fernandez-de-las-Penas C., Alburquerque-Sendin F. (2020). Influence of Clinical, Physical, Psychological, and Psychophysical Variables on Treatment Out-comes in Somatic Tinnitus Associated With Temporomandibular Pain: Evidence From a Randomized Clinical Trial. Pain Pract..

[38] Van der Wal A., Michiels S., Van de Heyning P., Braem M., Visscher C.M., Top-sakal V., Gilles A., Jacquemin L., Van Rompaey V., De Hertogh W. (2020). Treatment of Somatosensory Tinnitus: A Randomized Controlled Trial Studying the Effect of Orofacial Treatment as Part of a Multidisciplinary Program. J. Clin. Med..

[39] Lomas J., Gurgenci T., Jackson C., Campbell D. (2018). Temporomandibular Dysfunction. Aust. J. Gen. Pract..

[40] Hilgenberg P.B., Saldanha A.D.D., Cunha C.O., Rubo J.H., Conti P.C.R. (2012). Temporomandibular Disorders, Otologic Symptoms and Depression Levels in Tinnitus Patients. J. Oral Rehabil..

[41] Nascimento I.d.P., Almeida A.A., Diniz J., Martins M.L., de Freitas T.M.M.W.C., da Rosa M.R.D. (2019). Tinnitus Evaluation: Relationship between Pitch Matching and Loudness, Visual Analog Scale and Tinnitus Handicap Inventory. Braz. J. Otorhino-laryngol..

[42] Adamchic I., Langguth B., Hauptmann C., Alexander Tass P. (2012). Psychometric Evaluation of Visual Analog Scale for the Assessment of Chronic Tinnitus. Am. J. Audiol..

[43] Schaffer M., Bonel H., Sroka R., Schaffer P., Busch M., Reiser M., Dühmke E. (2000). Effects of 780 Nm Diode Laser Irradiation on Blood Microcirculation: Preliminary Findings on Time-Dependent T1-Weighted Contrast-Enhanced Magnetic Resonance Imaging (MRI). J. Photochem. Photobiol. B Biol..

[44] Chen C.H., Huang C.Y., Chang C.Y., Cheng Y.F. (2020). Efficacy of Low-Level Laser Therapy for Tinnitus: A Systematic Review with Meta-Analysis and Trial Sequential Analysis. Brain Sci..

[45] Tullberg M., Ernberg M. (2006). Long-Term Effect on Tinnitus by Treatment of Temporomandibular Disorders: A Two-Year Follow-up by Questionnaire. Acta Odontol. Scand..

[46] Jacquemin L., Mertens G., Van de Heyning P., Vanderveken O.M., Topsakal V., De Hertogh W., Michiels S., Van Rompaey V., Gilles A. (2019). Sensitivity to Change and Convergent Validity of the Tinnitus Functional Index (TFI) and the Tinnitus Questionnaire (TQ): Clinical and Research Perspectives. Hear. Res..

[47] Ebrahim S., Montoya L., Busse J.W., Carrasco-Labra A., Cuyatt C.H. (2012). The Effectiveness of Splint Therapy in Patients with Temporomandibular Disorders: A Systematic Review and Meta-Analysis. J. Am. Dent. Assoc..

[48] Racine M., Tousignant-Laflamme Y., Kloda L.A., Dion D., Dupuis G., Choinière M. (2012). A Systematic Literature Review of 10 Years of Research on Sex/Gender and Experimental Pain Perception—Part 1: Are There Really Differences between Women and Men?. Pain.

[49] La Touche R., Martínez García S., Serrano García B., Proy Acosta A., Adraos Juárez D., Fernández Pérez J.J., Angulo-Díaz-Parreño S., Cuenca-Martínez F., Par-is-Alemany A., Suso-Martí L. (2020). Effect of Manual Therapy and Therapeutic Exercise Ap-plied to the Cervical Region on Pain and Pressure Pain Sensitivity in Patients with Temporomandibular Disorders: A Systematic Review and Meta-Analysis. Pain Med..

[50] Calixtre L.B., Moreira R.F.C., Franchini G.H., Alburquerque-Sendín F., Oliveira A.B. (2015). Manual Therapy for the Management of Pain and Limited Range of Motion in Subjects with Signs and Symptoms of Temporomandibular Disorder: A Systematic Re-view of Randomised Controlled Trials. J. Oral Rehabil..

[51] Armijo-Olivo S., Pitance L., Singh V., Neto F., Thie N., Michelotti A. (2016). Effectiveness of Manual Therapy and Therapeutic Exercise for Temporomandibular Disorders: Systematic Review and Meta-Analysis. Phys. Ther..

[52] Bialosky J.E., Beneciuk J.M., Bishop M.D., Coronado R.A., Penza C.W., Simon C.B., George S.Z. (2018). Unraveling the Mechanisms of Manual Therapy: Modeling an Approach. J. Orthop. Sports Phys. Ther..

[53] Simons D.G. (2002). Understanding Effective Treatments of Myofascial Trigger Points. J. Bodyw. Mov. Ther..

[54] Hou C., Tsai L., Cheng K., Chung K., Hong C. (2002). Immediate Effects of Various Physical Therapeutic Modalities on Cervical Myofascial Pain and Trigger-Point Sensitivity. Arch. Phys. Med. Rehabil..

[55] Shimada A., Ishigaki S., Matsuka Y., Komiyama O., Torisu T., Oono Y., Sato H., Naganawa T., Mine A., Yamazaki Y. (2019). Effects of Exercise Therapy on Painful Temporomandibular Disorders. J. Oral Rehabil..

[56] Häggman-Henrikson B., Liv P., Ilgunas A., Visscher C.M., Lobbezoo F., Durham J., Lövgren A. (2020). Increasing Gender Differences in the Prevalence and Chronification of Orofacial Pain in the Population. Pain.

[57] Louw A., Zimney K., Puentedura E.J., Diener I. (2016). The Efficacy of Pain Neuroscience Education on Musculoskeletal Pain: A Systematic Review of the Literature. Physiother. Theory Pract..

[58] Langguth B., Kreuzer P.M., Kleinjung T., De Ridder D. (2013). Tinnitus: Causes and Clinical Management. Lancet Neurol..

