# Supplementary material for: Effectiveness of Physical Therapy Interventions for Temporomandibular Disorders Associated with Tinnitus: A Systematic Review
Source: J Clin Med. 2023 Jun 28;12(13):4329. doi: 10.3390/jcm12134329 (PMC10342521; doi:10.3390/jcm12134329)
Supplement: Supplementary file 1 [file jcm-12-04329-s001.zip › jcm-2306402-supplementary.pdf]

Supplementary material 1

Search strategy

MEDLINE (via OVID)

1. exp Temporomandibular Joint Disorders/ or Temporomandibular Joint Disorders.mp. – 18.937
2. exp Craniomandibular Disorders/ or Craniomandibular Disorders.mp. – 18.801
3. exp Facial Pain/ or Facial Pain.mp. – 12.428
4. exp Temporomandibular Joint Dysfunction Syndrome/ or Temporomandibular Joint Dysfunction Syndrome.mp. – 4.999
5. exp Myofascial Pain Syndromes/ or Myofascial Pain Syndromes.mp – 6.887
6. exp Temporomandibular Joint/ or Temporomandibular Joint.mp. or (exp Temporomandibular Joint Disc/ or Temporomandibular Joint Disc.mp.) – 29.763
7. syndrome, tmj.mp - 10
8. (costen syndrome or costen's syndrome or costens syndrome or syndrome, costen's).mp. - 94
9. exp Tinnitus/ or Tinnitus.mp – 15.193
10. exp Hearing Loss/ or Hearing Loss.mp. – 97.433
11. exp Ear Diseases/ or Ear Diseases.mp. – 170141
12. (ringing buzzing tinnitus or ringing-buzzing-tinnitus).mp. – 0
13. clicking tinnitus.mp. – 23
14. subjective tinnitus.mp. - 607
15. **POPULATION = 228.110**
16. exp Physical Therapy Modalities/ or Physical Therapy Modalities.mp. – 173.750
17. (physical therapy technique or physical therapy techniques).mp. - 141
18. exp Physical Therapy Specialty/ or Physical Therapy Specialty.mp. – 3.142
19. exp Rehabilitation/ or Rehabilitation.mp. – 588.373
20. exp Exercise Therapy/ or Exercise Therapy.mp. – 62.863
21. exp Telerehabilitation/ or Telerehabilitation.mp. – 1.880
22. exp Exercise Movement Techniques/ or Exercise Movement Techniques.mp. – 9.920
23. exp Musculoskeletal Manipulations/ or Musculoskeletal Manipulations.mp. – 18.403
24. exp Therapy, Soft Tissue/ or Therapy, Soft Tissue.mp. or (exp Manipulation, Chiropractic/ or Manipulation, Chiropractic.mp.) or (exp Kinesiology, Applied/ or Kinesiology, Applied.mp.) or (exp Manipulation, Osteopathic/ or Manipulation, Osteopathic.mp.) or (exp Dry Needling/ or Dry Needling.mp.) or (exp Cupping Therapy/ or Cupping Therapy.mp.) or (exp Laser Therapy/ or Laser Therapy.mp.) or (exp Electric Stimulation Therapy/ or Electric Stimulation Therapy.mp.) – 170.785

25. exp Cryotherapy/ or Cryotherapy.mp. – 32.911
26. exp Complementary Therapies/ or Complementary Therapies.mp. – 241.714
27. exp Exercise Therapy/ or Exercise Therapy.mp. – 62.863
28. exp Myofunctional Therapy/ or Myofunctional Therapy.mp. – 566
29. exp Manipulation, Spinal/ or Manipulation, Spinal.mp. – 1.828
30. exp Manipulation, Orthopedic/ or Manipulation, Orthopedic.mp. – 3.992
31. exp Transcranial Direct Current Stimulation/ or Transcranial Direct Current Stimulation.mp. – 7.057
32. exp Low-Level Light Therapy/ or Low-Level Light Therapy.mp. – 7.117
33. exp Counseling/ or Counseling.mp. – 120.237
34. **INTERVENTION – 1.064.248**
35. exp "Randomized Controlled Trials as Topic"/ or "Randomized Controlled Trials as Topic".mp. – 162.568

TOTAL = 233 articles

EMBASE

1. 'temporomandibular joint disorder'/exp - 16,392
2. 'face pain'/exp – 12,636
3. 'myofascial pain'/exp – 8,591
4. 'temporomandibular joint'/exp OR 'temporomandibular joint disc'/exp - 16,207
5. 'tmj syndrome' – 99
6. 'tinnitus'/exp – 24.270
7. 'hearing impairment'/exp – 136.977
8. 'ear disease'/exp – 171.680
9. 'ringing-buzzing-tinnitus' – 0
10. 'clicking tinnitus' – 27
11. 'clicking tinnitus' – 667
12. **POPULATION = 309,271**
13. 'physiotherapy'/exp – 106.870
14. 'physical therapy techniques' – 242
15. 'physical therapy specialty' – 282
16. 'rehabilitation'/exp – 470.791
17. 'kinesiotherapy'/exp – 96.893
18. 'telerehabilitation'/exp – 1.983
19. 'musculoskeletal manipulation'/exp – 4.866

20. 'soft tissue therapy'/exp – 172
21. 'chiropractic manipulation'/exp – 353
22. 'kinesiology'/exp – 2.204
23. 'osteopathic manipulation'/exp – 587
24. 'dry needling'/exp – 618
25. 'cupping therapy'/exp – 479
26. 'laser therapy'/exp – 28.826
27. 'electrotherapy'/exp – 293.543
28. 'cryotherapy'/exp – 58.165
29. 'alternative medicine'/exp – 73.061
30. 'muscle training'/exp – 13.566
31. 'spine manipulation'/exp – 964
32. 'orthopedic manipulation'/exp – 2.579
33. 'transcranial direct current stimulation'/exp – 9.624
34. 'low level laser therapy'/exp – 26.724
35. 'counseling'/exp – 194.394
36. **INTERVENTIONS = 1,220,100**
37. 'randomized controlled trial (topic)'/exp – 235.250

TOTAL = 366 artigos

CINAHL

1. (MH "Temporomandibular Joint Diseases+") - 5,643
2. (MH "Facial Pain+") - 3,404
3. (MH "Temporomandibular Joint Syndrome") – 665
4. "TMJ Syndrome" – 4,699
5. "Costen's Syndrome" - 4,696
6. "Orofacial Pain" – 1,157
7. "Neuralgic Facial Pain" – 1
8. "Craniofacial Pain" – 119
9. "Myofacial Pain" – 30
10. (MH "Tinnitus") – 5,522
11. "Somatic tinnitus" – 32
12. "Ringing-Buzzing-Tinnitus" – 3,958
13. "Clicking Tinnitus" – 14
14. "Somatosensory tinnitus" – 18

15. "Subjective tinnitus" – 199
16. **POPULATION = 13,978**
17. (MH "Physical Therapy+") – 158,935
18. "Physical therapy techniques" – 83
19. (MH "Rehabilitation+") – 319,196
20. "Mouth rehabilitation" - 112
21. (MH "Therapeutic Exercise+") – 61,806
22. "Exercise movement techniques" – 17,938
23. (MH "Manual Therapy+") OR (MH "Manipulation, Osteopathic") OR (MH "Manipulation, Chiropractic") OR (MH "Manipulation, Orthopedic") – 50,071
24. (MH "Myofascial Release") – 1,002
25. (MH "Electric Stimulation+") OR (MH "Electrical Stimulation, Functional") OR (MH "Transcutaneous Electric Nerve Stimulation") – 20,551
26. (MH "Transcranial Direct Current Stimulation") – 1,040
27. (MH "Photobiomodulation Therapy") – 59
28. (MH "Counseling+") – 42,361
29. **INTERVENTIONS = 383,857**
30. (MH "Randomized Controlled Trials+") – 132,379
31. (MH "Placebos") – 13,481
32. (MH "Clinical Trials+") – 342,438
33. **DESIGN OF STUDIES = 344,856**

TOTAL = 214 articles

CENTRAL

1. MeSH descriptor: [Temporomandibular Joint Disorders] explode all trees - 881
2. MeSH descriptor: [Facial Pain] explode all trees - 811
3. MeSH descriptor: [Temporomandibular Joint Dysfunction Syndrome] explode all trees - 360
4. (Face Pain):ti,ab,kw – 3.816
5. (Orofacial Pain):ti,ab,kw - 358
6. (Craniofacial Pain):ti,ab,kw - 118
7. ("myofacial pain"):ti,ab,kw - 47
8. MeSH descriptor: [Tinnitus] explode all trees - 698
9. (Somatic tinnitus):ti,ab,kw - 32
10. (Somatosensory tinnitus):ti,ab,kw - 31
11. ("subjective tinnitus"):ti,ab,kw - 200

12. **POPULATION = 6317**
13. MeSH descriptor: [Physical Therapy Modalities] explode all trees – 29.835
14. (Physical therapy techniques):ti,ab,kw - 2.537
15. ("physical therapy"):ti,ab,kw – 11.312
16. MeSH descriptor: [Rehabilitation] explode all trees - 41.137
17. MeSH descriptor: [Mouth Rehabilitation] explode all trees - 11
18. MeSH descriptor: [Exercise Therapy] explode all trees – 16.366
19. MeSH descriptor: [Exercise Movement Techniques] explode all trees - 2.540
20. MeSH descriptor: [Musculoskeletal Manipulations] explode all trees – 3.367
21. (Manual therapy):ti,ab,kw – 11.559
22. MeSH descriptor: [Manipulation, Chiropractic] explode all trees - 139
23. MeSH descriptor: [Manipulation, Orthopedic] explode all trees - 265
24. MeSH descriptor: [Manipulation, Spinal] explode all trees - 433
25. MeSH descriptor: [Myofascial Release Therapy] explode all trees - 14
26. MeSH descriptor: [Electric Stimulation Therapy] explode all trees – 8.009
27. MeSH descriptor: [Transcranial Direct Current Stimulation] explode all trees – 1.044
28. MeSH descriptor: [Low-Level Light Therapy] explode all trees – 1.167
29. MeSH descriptor: [Counseling] explode all trees – 6.008
30. **INTERVENTIONS = 70040**
31. MeSH descriptor: [Randomized Controlled Trial] explode all trees - 118
32. MeSH descriptor: [Clinical Trial] explode all trees - 141
33. **DESIGN = 141**

TOTAL = 749 articles

PEDro

1. Temporomandibular joint disorders – 79
2. Facial Pain – 56
3. TMJ Syndrome – 5
4. Temporomandibular Joint Syndrome – 16
5. Face Pain – 95
6. Orofacial Pain – 31
7. Craniofacial Pain – 7
8. Myofacial Pain – 14
9. Tinnitus – 48

TOTAL = 3 articles
